# Occupant safety effectiveness of proactive safety seat in autonomous emergency braking

**DOI:** 10.1038/s41598-022-09842-1

**Published:** 2022-04-06

**Authors:** Myeongkwan Kang, Hyungjoo Kim, Youngkuen Cho, Seonglae Kim, Dohyung Lim

**Affiliations:** 1 Department of Mechanical Engineering, Sejong University, Seoul, 05006 Korea; 2 Automotive Research and Development Division, Hyundai Motor Group, Hwaseong, 18280 Korea

**Keywords:** Mechanical engineering, Engineering, Biomedical engineering

## Abstract

The proactive safety seat (PSS) is a recently developed active safety system for securing occupant safety in out-of-seat position (OOSP), which was applied in the Hyundai Genesis G80 in 2020. However, there has not been sufficient quantifiable verification supporting the effectiveness of the PSS. The present study was performed to determine the effectiveness of the PSS for occupant safety in OOSP and to identify areas for additional improvement. Six test conditions were considered to determine the effectiveness of the PSS for augmentation of occupant safety in OOSP. Ten healthy men participated in the tests. Compared with the no PSS condition, maximum head excursion and neck rotation were significantly decreased in the PSS condition by 0.6–0.8-fold and 0.6–0.7-fold, respectively (*P* < 0.05). The PSS condition in which the seat pan was moved forward to the mid position showed a greater effect in reducing the characteristic motions related to submarining, compared with the condition in which the seat pan was moved rearward to the mid position (*P* < 0.05). These results suggested that PSS augments occupant safety in OOSP. This study provides valuable insights in ameliorating risks to the occupant in unintended seat positions before braking and/or collision.

## Introduction

Advanced driver assistance systems are technologies to improve vehicle safety. Autonomous emergency braking (AEB) is an active advanced driver assistance system that has been successfully implemented to improve vehicle safety since the 2000s^1^. Since its commercial development, AEB has been continuously tested and verified by the European New Car Assessment Program and National Highway Traffic Safety Administration^2,3,3–5^; it has been shown to prevent fatalities and serious injuries in collisions^6,7^. Furthermore, the National Highway Traffic Safety Administration and Insurance Institute for Highway Safety announced that all vehicles produced by the 20 major automotive manufacturers must be equipped with AEB by September 1, 2022^8^.

The protective capability of restraint systems is greatest when the occupants and their surroundings are in the normal seated position (NSP) at the start of the impact. For this reason, most previous studies of AEB set the occupant’s posture as the NSP. AEB has been reported to reduce the rates of front-impact collisions by 27%, rear-end collisions by 27–50%, and injuries from rear-end collisions by 35–56%^9,10^. Although AEB effectively reduces collisions, however, on the other viewpoint, it means that the possibility of collisions remains above 50%. Moreover, real-world accident field data show that in many instances, especially when a severe injury occurs, both seats and occupants are not in the designed position at the time of impact. This is presumably because the occupant selects a comfort-emphasizing seating position. Even though the NSP is the standard seating condition in crash tests, this condition has limitations for understanding the effects on the occupant in an out-of-seat position (OOSP)^11–14^.

The occupant’s muscular activation characteristics in the OOSP during sudden braking or collision circumstances have been suggested considerably in previous studies. Brault et al. reported that if a seat is absent to proper position, greater muscle strains would likely develop, escalating the potential for injurious lengthening muscle contractions^15^. Horst et al. and Vekataramana et al. reported that the initial seating posture strongly influences human motion, and an improper seating posture may increase the probability of the high muscular contraction created by the increased body motion^16,17^. Kang et al. indicated that occupants’ maximum tension in the OOSP of the sternocleidomastoid muscle and the rectus abdominis muscle increased 3.8 ± 0.1-fold and 2.9 ± 0.2-fold than that for in NSP, respectively^14^. These previous studies showed the existence of a high correlation between the increase of muscular contraction and occupant’s seat conditions. However, research has not been revealed sufficiently to ensure the occupant’s safety in OOSP.

To ensure occupant safety in OOSP, previous studies outlined the requirements for active safety systems that change the seat configuration before the event of a forward/rear collision or sudden braking/turning^14,18–23^. In 1996, Saab, Mercedes Benz, BMW, Toyota, and Volvo introduced passive safety seat mechanisms with proactive head restraints and reactive seats^18^. Daimler Chrysler, BMW, Toyota, and Transport Research Laboratory reported the concept of adjusted seat position, which shifts the seat position from forward to the rear before impact^19,21–23^. Transport Research Laboratory performed several sled tests and reported supplementary effects on occupant safety related to the reduction of neck rotation, chest excursion, and/or pelvic excursion when the seat was moved forward from the rear. The Transport Research Laboratory sled test results demonstrated augmentation of occupant safety with a front adjusting seat pan, but these tests were only performed using 5th and 50th percentile male anthropomorphic test devices; they did not consider OOSP or quantify seat control strategies. Furthermore, Daimler Chrysler, BMW, and Toyota presented only conceptual reports without showing an actual device. In 2020, Hyundai Motor Company acquired a patent for a proactive adjustment safety control system; it applied the proactive safety seat (PSS) in the Genesis G80^24^. The main function of the PSS is to adjust the position of the seat pan and the angle of the seat back when the possibility of a collision exceeds the expected value, placing the seat in a predetermined state when the seat position or angle of the seat back is not in the intended position. Theoretically, the PSS is an ideal safety assistance system for occupants in an OOSP. However, because of its relative novelty, there has not been sufficient quantifiable verification supporting the effectiveness of this system.

This study was performed to determine the effectiveness of the PSS for occupant safety in OOSP and to identify possible improvements. To improve PSS, tests were performed to confirm the effects of moving the seat pan forward or rearward to the rear during seat back straightening before AEB operation.

## Results

### Characteristics of motion responses during PSS operation

**Figure 1:**
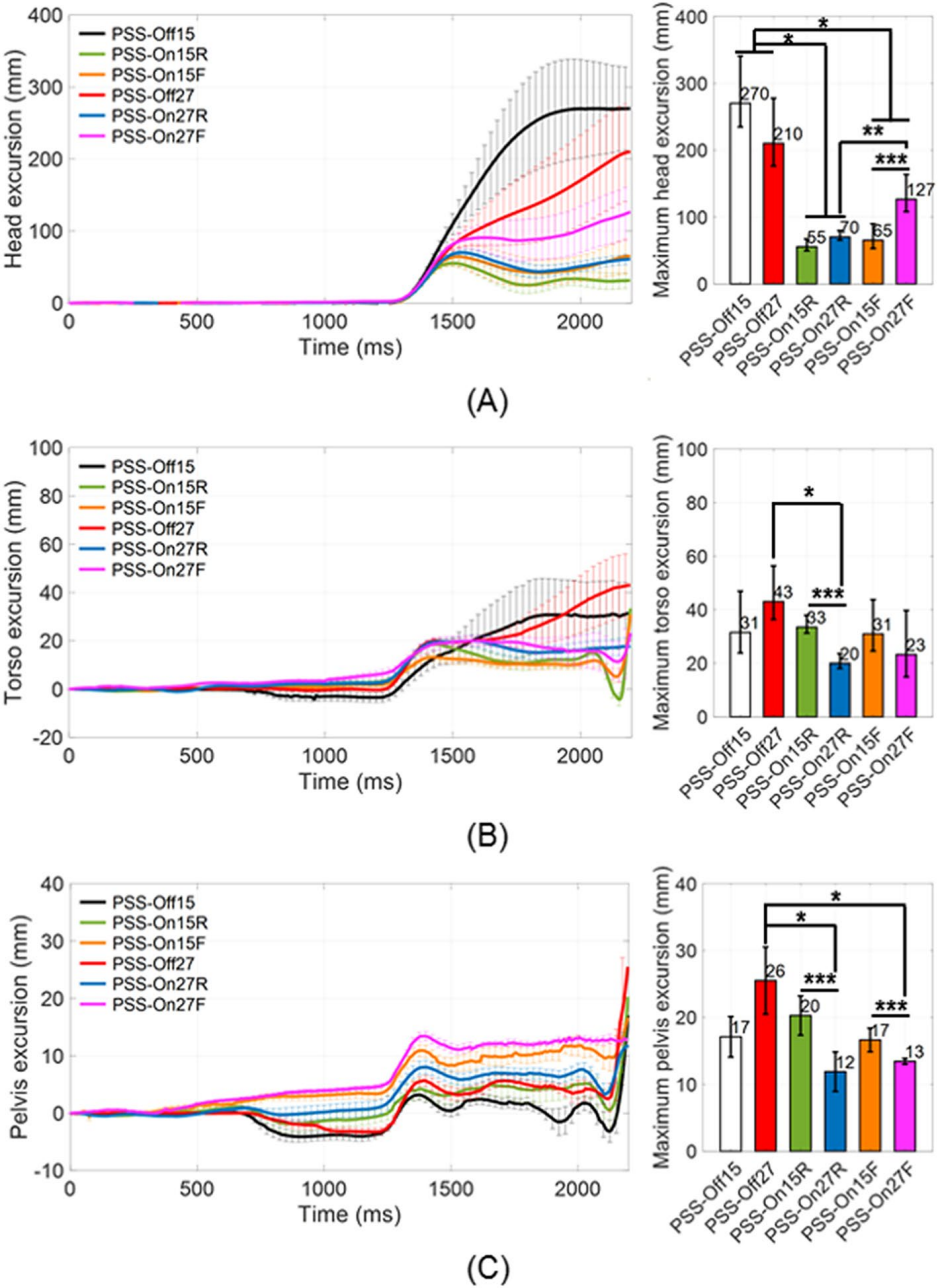
Time-dependent mean excursions of random five volunteers among the total in the sagittal (x–z) plane for (**A**) head, (**B**) torso, and (**C**) pelvis (*P < 0.05 for comparison of PSS application; ** P < 0.05 for comparison of seat pan movement modes in PSS; *** P < 0.05 for comparison of initial seat back angles).

**Figure 2:**
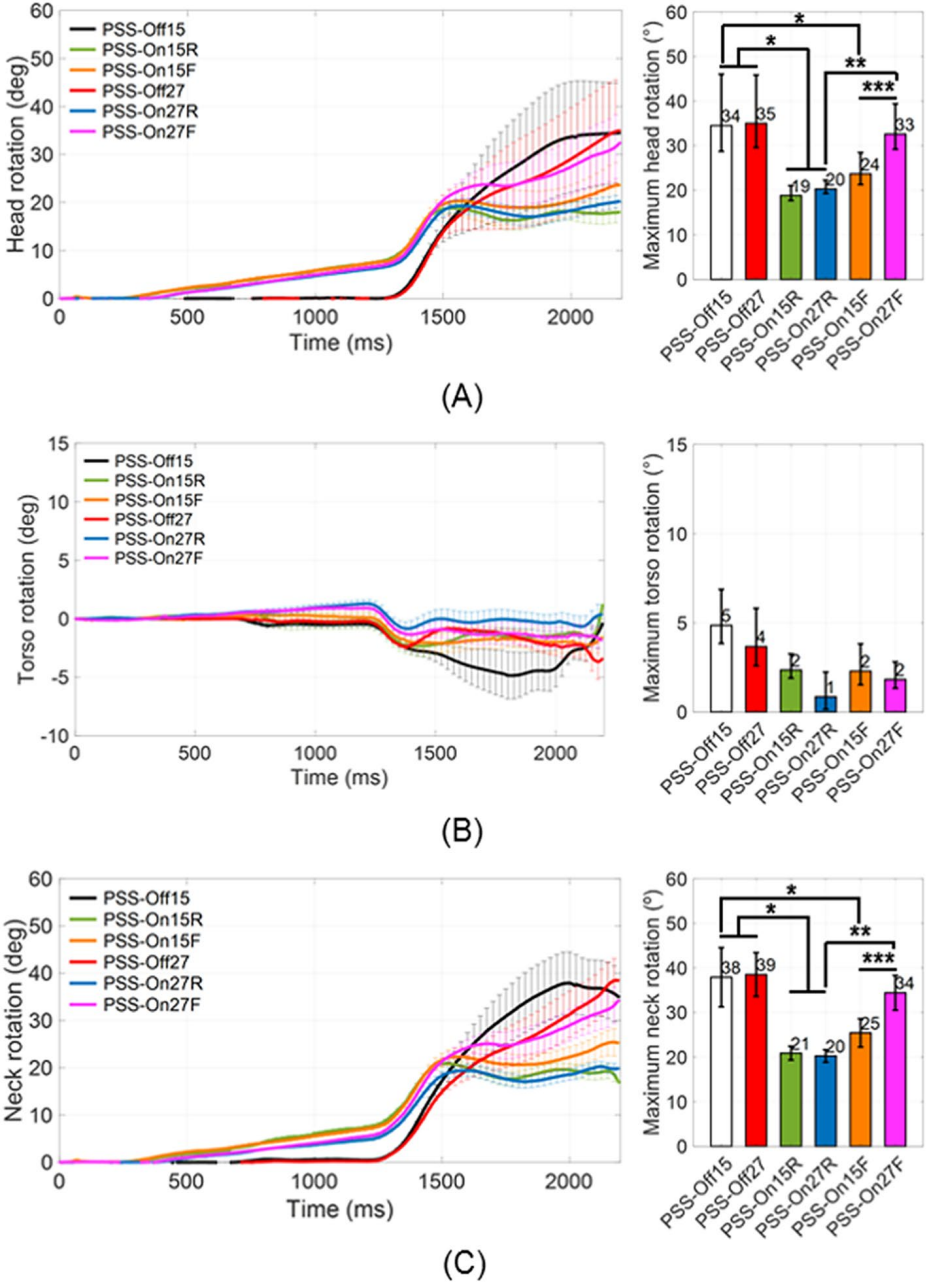
Time-dependent mean excursions of random five volunteers among the total in the sagittal (x–z) plane for (**A**) head, (**B**) torso, and (**C**) neck (*P < 0.05 for comparison of PSS application; **P < 0.05 for comparison of seat pan movement modes in PSS; ***P < 0.05 for comparison of initial seat back angles).

Figures 1 and 2 show the time-dependent excursions of the head, torso, pelvis, as well as rotations of the head, torso, and neck in the sagittal (x–z) plane, for each test condition. There were no significant differences in all regions’ excursion or rotation between PSS Off with 15° reclined seat angle (PSS_Off15) and PSS Off with 27° reclined seat angle (PSS_Off27) (*P* > 0.05). Moreover, these conditions had the biggest standard errors among test conditions. PSS_Off15 and PSS_Off27 had maximum head excursions of 270.2 ± 70.8 mm and 210 ± 67.7 mm, respectively; maximum torso excursions of 31.5 ± 15.4 mm and 43.0 ± 13.4 mm, respectively; maximum pelvic excursions of 17.1 ± 6.0 mm and 25.5 ± 10.0 mm, respectively; maximum head rotations of 34.5° ± 11.5° and 35.0° ± 10.9°, respectively; and maximum neck rotations of 37.9° ± 7.4° and 38.5° ± 9.0°, respectively.

In PSS On and the seat pan moved rearward to the mid position with 15° reclined seat angle (PSS_On15R) and PSS On and the seat pan moved rearward to the mid position with 27° reclined seat angle (PSS_On27R), the maximum head excursion, head rotation, and neckrotationwere significantly decreased than those values in PSS_Off. There were significant differences in the torso and pelvis excursions between PSS_On15R and PSS_On27R (*P* < 0.05), and the maximum torso and pelvis excursions in PSS_On15R were greater 13.5 ± 3.2 mm and 8.4 ± 2.9 mm than in PSS_On27R respectively. Additionally, the occupant motion characteristics were generally uniform and showed the standard errors lower than those values in PSS_Off. The maximum head excursions were 55.4 ± 11.7 mm and 70.1 ± 9.4 mm, respectively; these were 216 ± 14.5 mm and 147 ± 13.5 mm less than in PSS_Off15 and PSS_Off27, respectively (*P* < 0.05). The maximum head rotations were 18.8° ± 2.2° and 20.2° ± 1.9°, respectively; these were 15.7° ± 4.3° and 14.8° ± 3.1° less than in PSS_Off15 and PSS_Off27, respectively (*P* < 0.05). The maximum neck rotations were 20.9° ± 1.7° and 20.3° ± 0.2°, respectively; these were 15.2° ± 0.1° and 19.3° ± 0.1° less than in PSS_Off15 and PSS_Off27, respectively (*P* < 0.05). On the other hand, the significant differences for the torso and pelvis excursions had been indicated solely in PSS_On27R that were compared with PSS_Off27 (*P* < 0.05). The maximum torso excursion in PSS_On27R was 19.9 ± 3.6 mm, which was 23.1 ± 2.7 mm less than in PSS_Off27 (*P* < 0.05). The maximum pelvic excursion in PSS_On27R was 11.9 ± 2.9 mm, which was 13.6 ± 3 mm less than in PSS_Off27 (*P* < 0.05).

In PSS On and the seat pan moved forward to the mid position with 15° reclined seat angle (PSS_On15F) and PSS On and the seat pan moved forward to the mid position with 27° reclined seat angle (PSS_On27F), the maximum head excursions were significantly decreased, compared with those values in PSS_Off. There were significant differences in the head and pelvis excursions and the head and neck rotation between PSS_On15F and PSS_On27F (*P* < 0.05). The maximum head excursion, head rotation, and neck rotation in PSS_On27F were higher 61.3 ± 30.1 mm, 8.9° ± 5.7°, and 9.0° ± 4.7° than those for PSS_On15F, respectively. Whereas the maximum pelvis excursion in PSS_On15F was greater 3.2 ± 1.1 mm than that for PSS_On27F. The occupant motion characteristics in PSS_On15F and PSS_On27F were generally uniform and showed the standard errors lower than those values in PSS_Off. The maximum head excursions in PSS_On15F and PSS_On27F were 65.2 ± 24.5 mm and 126.5 ± 37.0 mm, respectively; these were 216 ± 14.2 mm and 63 ± 13.5 mm less than in PSS_Off15 and PSS_Off27, respectively (*P* < 0.05). The pelvic excursion in PSS_On27F, and head and neck rotation in PSS_On15F showed significant differences independently (*P* < 0.05). The maximum pelvic excursion in PSS_On27F was 13.4 ± 0.9 mm, which was 12.1 ± 2.1 mm less than in PSS_Off27 (*P* < 0.05). The maximum head and neck rotation in PSS_On15F was 23.6° ± 4.7° and 25.4° ± 3.6°, which were 10.8° ± 5.7° and 11.4° ± 0.7° less than in PSS_Off15 respectively (*P* < 0.05). In addition, by the results in comparison of the initial seat back angles, the maximum head excursion in PSS_On27F was 0.6 ± 0.3-fold greater than in PSS_On27R (*P* < 0.05). The maximum head and neck rotations in PSS_On27F were 0.6 ± 0.1-fold and 0.4 ± 0.3-fold greater than in PSS_On27R and PSS_On15F, respectively (*P* < 0.05).

### Characteristics of shoulder and belt tension during PSS operation

**Figure 3:**
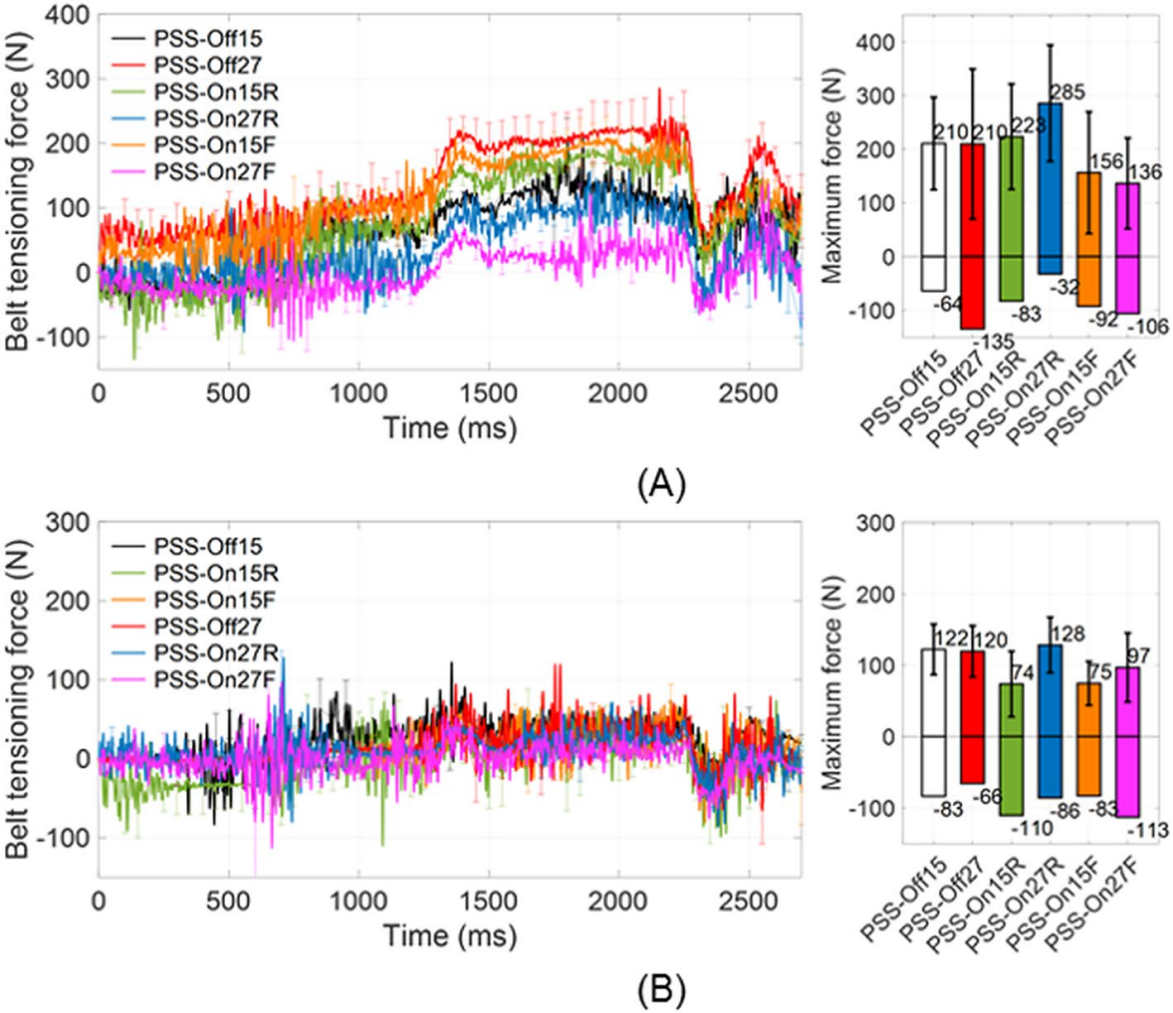
Time-dependent mean excursions of random five volunteers among the total for (**A**) shoulder and (**B**) lap belts.

Figure 3 shows the time-dependent tensioning forces in the shoulder and lap belts for each test condition. The maximum shoulder belt tensions increased after 1200 ms that initiating time of the accelerating sled, but there were no significant differences in all test conditions (*P* > 0.05). Additionally, the activation of shoulder and lap belts forces typically occurred between 1200 and 1500 ms. However, in PSS_On15F and PSS_On27F, the lap belt activation occurred at approximately 600 ms when PSS was operating; their maximum shoulder belt tensions were lower than those for the other conditions.

### Characteristics of muscle activity during PSS operation

**Figure 4:**
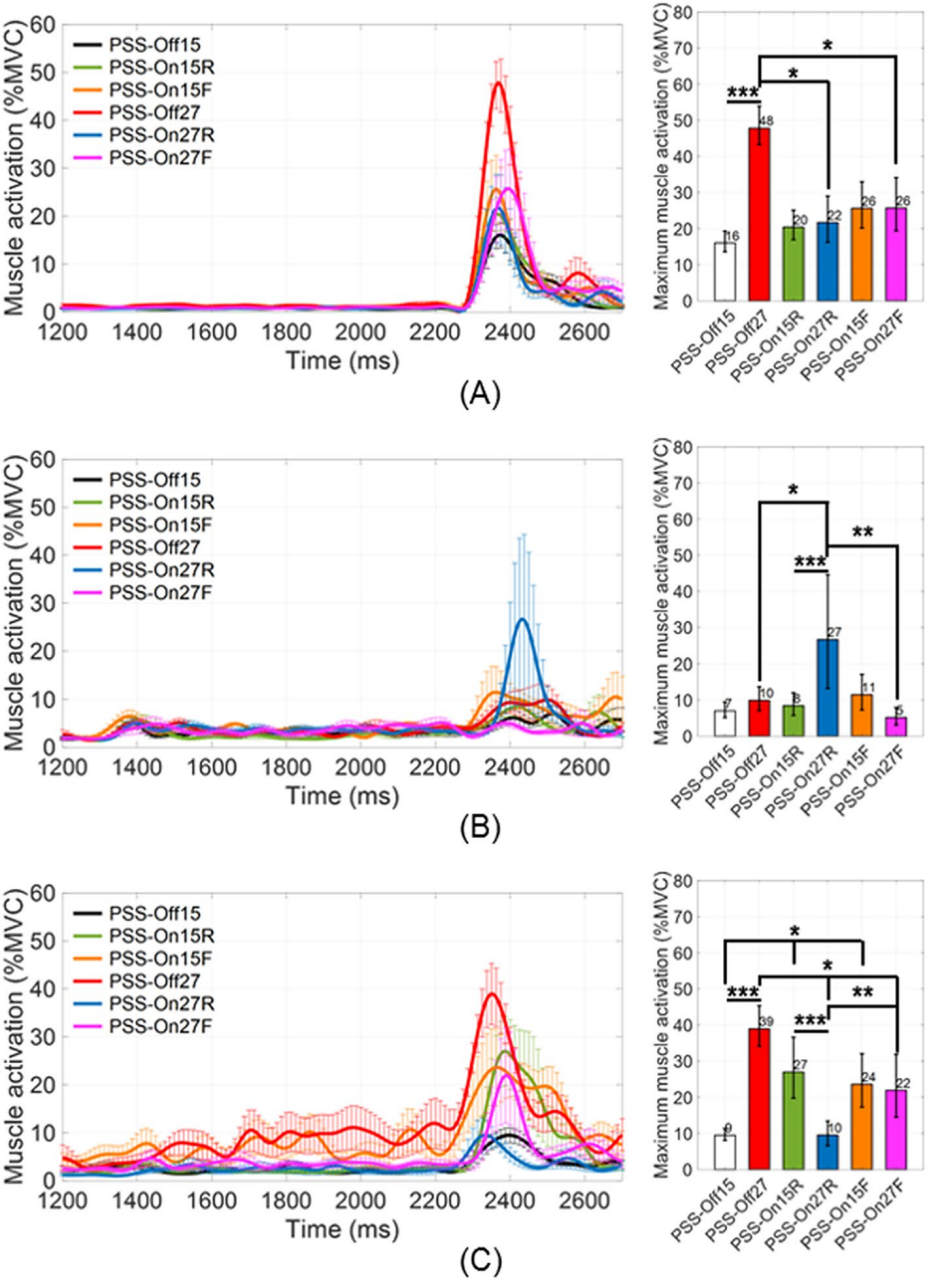
Time-dependent mean excursions of random five volunteers among the total for (**A**) left SCM, (**B**) right splenius, and (C) right longissimus (*P < 0.05 for comparison of PSS application; **P < 0.05 for comparison of seat pan movement modes in PSS; ***P < 0.05 for comparison of initial seat back angles).

Figure 4 shows the maximum activities of the major muscles for each test condition. The maximum muscle activities showed remarkably significant differences in solely three muscles: left sternocleidomastoid (SCM) muscle, right splenius muscle, and right longissimus muscle (*P* < 0.05). Maximum left SCM muscle activity in PSS_Off27 was 47.8% ± 6.1% maximal voluntary contraction (MVC); maximum left SCM muscle activity in PSS_On27R was 21.7% ± 7.3%MVC, which was 0.5 ± 0.2-fold lesser than in PSS_Off27 (*P* < 0.05). In PSS_On27F, it was 25.7% ± 8.4%MVC, which was 0.5 ± 0.3-fold lesser than in PSS_Off27 (*P* < 0.05). By the results in comparison of the initial seat back angles, maximum muscle activation in PSS_Off27 was greater 31.8% ± 3.6%MVC than that for PSS_Off15. Maximum right splenius muscle activity in PSS_Off27 was 9.8% ± 3.8%MVC; maximum right splenius muscle activity in PSS_On27R was 26.6% ± 17.9%MVC, which was 1.7 ± 8.1-fold greater than in PSS_Off27 (*P* < 0.05). Furthermore, by the results in comparison of the seat pan movement modes in PSS, maximum muscle activation in PSS_On27R was greater 21.5% ± 9.4%MVC than PSS_On27F. By the results in comparison of the initial seat back angles, maximum muscle activation in PSS_On27R was greater 18.2% ± 8.9%MVC than that for PSS_On15R. Maximum right longissimus muscle activity in PSS_Off15 and PSS_Off27 were 9.5% ± 1.9%MVC and 38.9% ± 6.4%MVC respectively; maximum right longissimus muscle activity in PSS_On15R and PSS_On15F were 27.0% ± 9.6%MVC and 23.6% ± 8.4%MVC, which were 1.8 ± 4.4-fold and 1.5 ± 3.8-fold greater than in PSS_Off15 (*P* < 0.05). In PSS_On27R and PSS_On27F, maximum muscle activations were 9.5% ± 3.9%MVC and 21.9% ± 9.9%MVC, which were 0.8 ± 4.8-fold and 0.4 ± 8.3-fold lesser than in PSS_Off27 (*P* < 0.05). Additionally, by the results in comparison of the seat pan movement modes in PSS, maximum muscle activation in PSS_On27R was greater 12.4% ± 5.2%MVC than PSS_On27F. By the results in comparison of the initial seat back angles, maximum muscle activation in PSS_Off27 was greater 29.4% ± 3.3%MVC than that for PSS_Off15, and maximum muscle activation in PSS_On15R was greater 17.5% ± 6.4%MVC than that for PSS_On27R. On the other hand, the other muscle activity regions did not show significant differences (*P* > 0.05) and all remained below 20%MVC.

## Discussion

The results of the present study showed that PSS is effective as an active safety system for ensuring occupant safety by counteracting OOSP before AEB operation. In near future, occupants are likely to adopt various postures for comfort before partially or fully autonomous driving, but there have been insufficient data regarding the safety of occupants in such postures. Several experimental and/or finite element model studies were performed to examine the responses of the occupant in unintended seat positions^14,25,26^. These studies verified the jeopardy of undesigned seat positions, but they did not propose means of counteracting these perils for occupant safety. In the development of an active safety system, it is important to ensure occupant safety before braking and/or collision in haphazard seat positions. In addition, information regarding the characteristics of human responses during the operation of the safety system will allow safety improvement by controlling the interactions of active and passive safety systems. This study confirmed the effectiveness of PSS operation to improve the interactions of active/passive safety systems and determined the characteristics of human responses during the operation of the safety system. Therefore, this study provides valuable insights to ameliorate risk to the occupant in accidental seat positions before braking and/or collision.

In the two-stage braking maneuver for AEB operation, to operate a maximum braking acceleration of 1 g under 50 km/h, AEB decelerates the vehicle’s speed gradually from TTC 1.6 s^11,27^. Previous studies reported that AEB at TTC 1.6–0.6 s starts a deceleration of 0.2–0.6 g; this phase is defined as the partial braking phase^28^. Referring to the above AEB operating mechanism, the tests in this study performed the partial braking phase exhibiting acceleration to 0.4 g for 1,000 s to assess the effects of PSS operation. Whereas we assumed that continued adjustment of the seat position during AEB operation would lead to unpredictable conditions and increase endangerment to the occupant because of incomplete PSS operation. For this reason, PSS operation was performed in TTC 2.6 to 1.6 s, which is before the partial braking phase of AEB.

However, TTC is sole a theoretical concept and it can differ from real-world conditions; thus, there are enough likelihoods that occurrence of a collision before the maximum braking operation of AEB. To alter from the fully reclined seat to NSP, PSS required at least double the 1 s that of TTC 2.6 to 1.6 s. Because of the designed time algorithm for completing PSS operation within 1 s and verification of the test environment only in the partial braking phase, the present test results may have limits for fully considering PSS that any OOSP be adjusted to NSP. Withal, to our knowledge, this study represents the first attempt to determine the effectiveness of the PSS for counteracting risk to the occupant in OOSP. Thus, the limits of the PSS operation process in the tests could be regarded to consideration of that the occupant’s safety against unpredictable perils during incomplete PSS operation. Furthermore, the characteristics of occupant responses in the partial braking phase could be utilized to gain fundamental insights to facilitate the improvement of active safety systems, such as PSS strategies. From this viewpoint, the experimental parameters for the PSS were sufficiently covered, and our results can be considered both valid and reliable.

**Figure 5:**
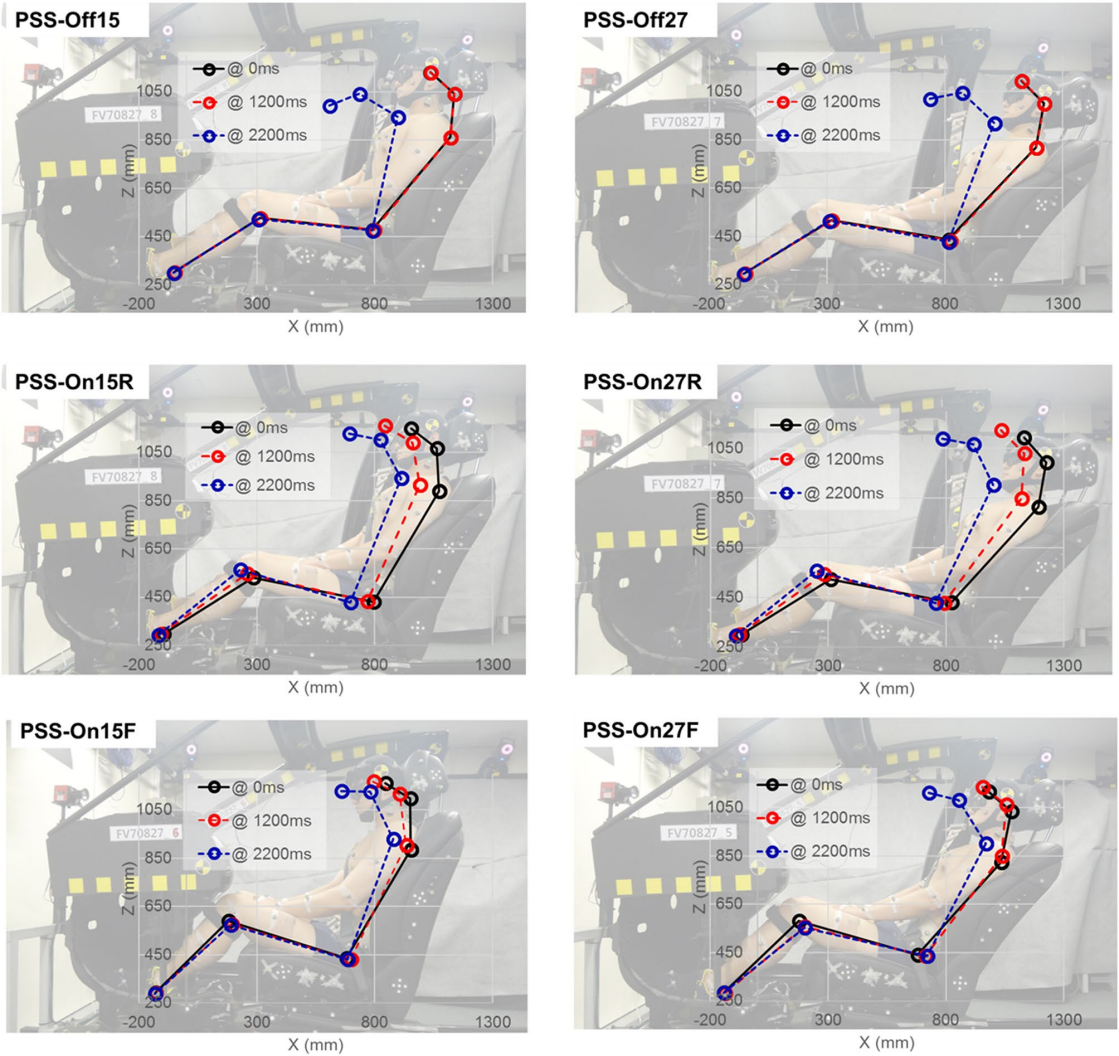
Frontal motion trajectories of an occupant in the sagittal (x–z) plane. Photographs were taken by Musculoskeletal Bioengineering Laboratory, Sejong University.

The understanding and utilization of the PSS operating mechanism can be beneficial to ensure occupant safety, adjusting both the seat and occupant’s posture to the intended design position before the time of a collision. The protective operation of restraint systems is greatest when occupants and their ambient environment are in the intended design position (i.e., NSP) at the start of the collision^11–14^. However, real-world accident records indicate that, in many instances, both the seats and the occupants are not in the designed position at the time of a collision. Therefore, the functional mechanism of the PSS to adjust the seat position of the occupant to NSP before a collision is important to counterbalance the jeopardy by OOSP. The results of the present study showed that, regardless of whether the seat pan shifted forward or rearward, the occupant’s frontal motion with PSS operation was generally decreased and the motion characteristics showed a small standard error range, compared with PSS_Off conditions. In particular, PSS_On27R and PSS_On27F showed a decrease of the characteristic motions related to submarining (e.g., upper body hovering and falling, as well as pelvic slipping)^14^. These results indicated that the PSS had a protective effect when the occupant’s seat position was adjusted closer to NSP. However, the head excursion and neck rotation were 0.8 and 0.7-fold greater in PSS_On27F than in PSS_On27R, respectively. To confirm whether there were differences in PSS effectiveness because of postural adjustment between PSS_On27R and PSS_On27F, we compared the occupant’s postures at 1200 ms, which PSS operation was completed. As shown in Fig. 5, the changes in torso rotation angle by PSS operation in PSS_On27R and PSS_On27F were from 63.1° ± 0.4° to 68.0° ± 0.3° and from 63.7° ± 0.7° to 68.5° ± 0.6°, respectively; these were not markedly different. In contrast, the changes in head rotation angle in PSS_On27R and PSS_On27F were from 44.3° ± 1.8° to 40.0° ± 1.7° and from 41.0° ± 1.8° to 36.0° ± 1.5°, respectively; these represented a difference of approximately 5° between the two conditions. These results mean that PSS_On27R and PSS_On27F had different neck rotation angles in each initial posture depending on altered head rotation angle and head center of gravity. Occupants in PSS_On27R tended to lean more heavily on the headrest, which may have been because of the difference in center of gravity caused by the lower body posture and seat pan location. Furthermore, these differences led to pelvic-on-femoral osteokinematics. When the seat pan location moves rearward with the supralumbar trunk stationary on the seat back, the anterior pelvic tilt (flexion) will occur, and coincidentally the lumbar spine would likely have a larger curvature toward the forward. The human body motion is the summation that consists of the external force and internal force. Previous studies had mainly used the mass-spring-damper systems in representing the mechanical impedance of human body motion^29,30^.

1 $$ me(t)\ddot{x}(t) + be(t)\dot{x}(t) + ke(t)x(t) = fe(t) $$

Equation (1) is the mass-spring-damper model representing the joint torque. This is the second-order dynamic equation where *me*(*t*), *be*(*t*) and *ke*(*t*) the impedance parameters, which denote the mass, damping factor, and stiffness of joints, respectively; and fe(t) presenting the force exerted to joints. It means the human body motion depends on the joint stiffness and damping characteristics. Furthermore, the muscular structure and properties would likely significantly affect the degree of joint stiffness. However, the joint and muscle characteristics highly rely on their particular directions and states (e.g., muscle state of contraction/relaxation, joint state of flexion/extension, and so on). Hence, different occupant’s initial postures by seat pan moving directions of PSS would likely be the main factor that generates disparate motion characteristics during AEB. Additionally, to identify the regularity of muscular interaction, we compared the amounts of muscle activation for each PSS strategy. Although the motion and muscle characteristics can be different due to using what PSS strategy to reach the NSP before AEB operation, the test results indicate that most head and neck excursions decreased when applied PSS. Therefore, it may consider that PSS strategies serve meaningful effects to restrict more safely the occupant motion.

The maximum belt tensioning force on the shoulder and lap belt was not significantly different under each test condition (*P* > 0.05). On the other hand, the maximum muscular activities indicated significant differences diversely under each test condition (*P* < 0.05). These differences show that the possibility of muscle restraining activity in SCM was ameliorated, compared with the muscle activation in PSS_Off27. Furthermore, it means that characteristics of muscle activation-particularly longissimus muscles-would likely have a difference regarding each posture of an occupant. The muscle activities measured in all seat positions peaked at approximately 2400 ms after activation of partial braking, at approximately 100–200 ms after the excursions had reached their maximum values. This point would support the phenomena of damping force occurrences with respect to changing joint stiffness and muscle contraction. However, all muscle activities of the occupants were below 50% of the MVC values. These results may have been limited by the low-speed conditions-the partial braking phase of AEB-used in the tests. Hence, further research is needed to quantify the actual risk of muscle injury. In this study, although the muscle activities during PSS operation were not expected to reflect a risk of muscle injury, these results are valuable for improving the overall understanding of occupant motions during AEB with PSS.

Several factors that can directly or indirectly influence the responses of occupants during the tests (e.g., habituation, lack of awareness, and startle response) have been studied^14,20,31,32^. Blouin et al.^32^ noted the effects of habituation and acoustic startle stimulus of the test platform on resulting occupant responses. They suggested that it is important to perform tests without habituation and acoustic startle stimulus to clearly observe occupant responses. Beeman et al.^31^ studied the effects of contracted muscles on occupant kinematics and kinetics; they showed that muscle condition before the test had a significant effect on the occupant motion response. These studies suggested that effective exploration of experimental parameters would facilitate better characterization of occupant motion responses^14,20^. Therefore, although the objective of the present study was to examine the test parameters of the PSS that influence the results, comprehensive observation was not possible because of limitations regarding the test environment and conditions. The selection of PSS strategy was restrictive because of considerations regarding test volunteer safety. However, because the occupant motion characteristics for the OOSP measured here were similar to the characteristics reported in previous studies, the experimental parameters may have been sufficient and our results can be considered both reliable and valid^14^. The sample size and specific type of tested subjects selected in this study might be limitations to precisely attest the PSS effectiveness. However, the restrictive sample size was the situational limitation of this study, and the particular type of volunteer may be more beneficial to define the criterion of human models. If we verified with volunteers of more people and various types in the future study, we would get better results to identify the effects of PSS.

Furthermore, the results of this study are highly meaningful because we identified the effectiveness of PSS implementation during AEB operation. Importantly, the acquisition of human characteristics for PSS effectiveness in this study was novel and the test parameters were covered sufficiently; thus, our results can be considered reliable and valid. These results represent essential information regarding the improvement of safety device effectiveness to ameliorate occupant restraint and counteract any deterioration of occupant safety. Further studies are required to devise a PSS mechanism suitable for a more diverse range of emergency maneuvers, such as evasive swerving, to further improve occupant safety. This study provides a basis for obtaining a clear understanding of occupant motion responses for active PSS and for the identification of potential discomfort or injuries to the occupant during AEB operation.

Overall, the results of the present study indicated that the PSS can improve occupant safety in OOSP during AEB operation. The occupant’s frontal motion was generally decreased with PSS operation; the characteristics of frontal excursion showed a small range of standard error, compared with the PSS_Off condition. In the fully reclined seat position, PSS operation led to a reduction of the characteristic motion associated with submarining of the occupant.

In conclusion, PSS operation can improve the safety of occupants in OOSP. However, the occupant safety effects according to various PSS operation strategies must be further verified. To secure occupant safety with various PSS operation strategies, additional confirmation is required for more diverse emergency maneuvers, such as evasive swerving and/or the use of recent occupant restraint configurations (e.g., belt-in-seat components). Moreover, if the PSS control system can perform with better classification of the occupant’s condition (e.g., body type, awareness/lack of awareness, and upper body center of gravity) in autonomous vehicle driving, the optimized algorithm for PSS strategies may provide greater occupant safety improvements.

## Methods

### Volunteers

The test group consisted of 10 healthy men in their 20s who had no prior histories of cervical spine injury or degenerative changes. The selected volunteers were ± 2.5% for height and ± 1.5% for weight of the 50th percentile male hybrid III dummy (Table 1). Informed consent to publish was obtained from all the volunteers to include the images and information in the manuscript.

**Table 1:** Volunteer characteristics.

Test subject (Initial)	Height (mm)	Neck length (mm)	Back distance (mm)	Sitting height (mm)	Weight (kg)	BMI (kg/m^2)^	Leg length (mm)	Knee width (mm)
S1 (KJY)	1788	114	834	948	71.8	22.46	930	115
S2 (JYH)	1782	113	828	941	80.2	25.26	910	117
S3 (PMY)	1802	114	835	949	78.9	24.3	910	120
S4 (MHW)	1771	114	837	951	72.9	23.24	910	120
S5 (KMG)	1800	112	818	930	84	25.93	940	115
S6 (KCH)	1781	109	799	908	72.9	22.98	920	110
S7 (KJH)	1813	115	842	957	82.8	25.19	930	115
S8 (LGR)	1789	113	836	949	79.2	24.75	930	105
S9 (KSW)	1803	114	828	942	76.5	23.53	920	115
S10 (LSH)	1787	114	835	949	80	25.05	900	110
Avg	1792	113	829	942	78	24	920	114
S.E	1.26	0.45	3.60	1.41	0.43	0.12	1.25	0.47

### Test platform and configuration

**Figure 6:**
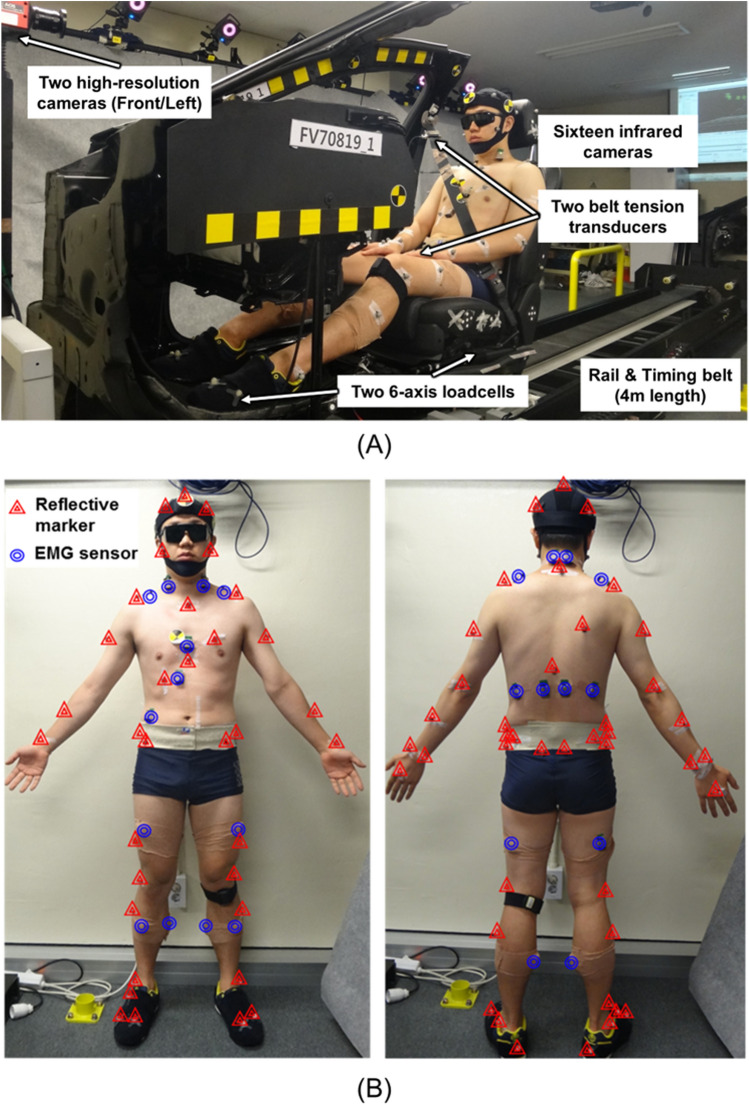
(**A**) PSS test platform. (**B**) Locations of the reflective markers and wireless surface EMG sensors. Photographs were taken by Musculoskeletal Bioengineering Laboratory, Sejong University.

**Figure 7:**
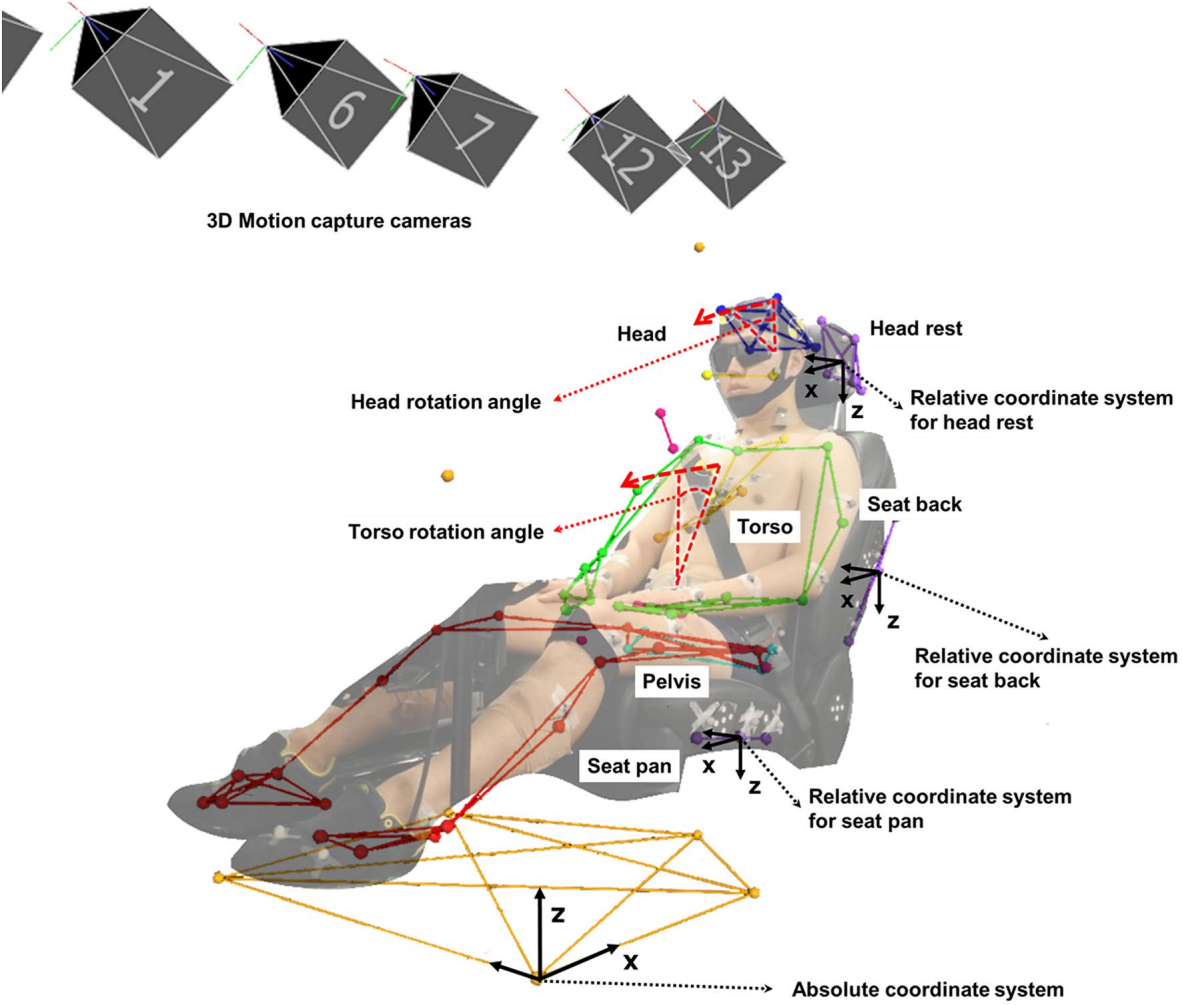
Definitions of head and torso angles and global and local coordinate systems, as well as the segment model constructed by connecting the markers with lines. The photograph was taken by Musculoskeletal Bioengineering Laboratory, Sejong University.

The test platform is shown in Figure 6 (A). The test buck was composed of a passenger seat from a Genesis EQ-900 (Hyundai Motor Group, Seoul, Korea). A servo motor (APM-FGP150GMK, 15 kW capacity, 1500 rpm; LS Mecapion, Gyeonggi-do, South Korea) was used to control the test environment. A three-dimensional motion capture system with 16 infrared cameras was used to measure occupant excursion with a sampling rate of 200 Hz (T-20s; Vicon Motion Systems Ltd., Oxford, UK). To track the excursion of the test volunteers, a customized marker set based on a general plug-in-gait set was used^31,33^. The excursions of body parts were measured with three virtual central markers that were determined by the following actual reflective markers: five at the head, seven at the torso, and eight at the pelvis. To define accurate measurement of the excursion, a global coordinate system was defined through the baseplate of the sled; local coordinate systems were defined by virtual central markers at the headrest, seat back, and seat pan (Figure 7). This mechanism allowed to measure the occupant’s partial body region (head, torso, pelvis) motion independently for which contacts with each part of the seat. To assist in the interpretation of excursion measured by the three-dimensional motion capture system, two high-resolution cameras (Q-MIZE HD v2, 500 fps; AOS Technologies, Dättwil, Switzerland) were installed at the front and left sides of the AEB test platform. To confirm the shift in center of gravity, two six-axial load cell sensors (CWW11-K100 & UMMA [100 kgf], sampling rate: 2 kHz; DACELL, Qingdao, China) were attached beneath the seat and footrest, respectively. Two seatbelt tension transducers (LBT-E, sampling rate: 2 kHz; Kyowa Electronic Instruments, Tokyo, Japan) were used to measure the shoulder and lap belt restraining forces. All kinetic measurements were filtered through a low-pass filter with channel frequency class 60 Hz, conforming to the SAE J211 filter standard^34,35^. To measure muscle activities of the test volunteers, a wireless surface electromyogram (EMG) (Tringo Wireless EMG System, sampling rate: 2 kHz; Delsys, Inc., Boston, MA, USA) was used in parallel with the three-dimensional motion capture system described above (Figure 6 (B)). The wireless surface EMG was attached to the neck joint region (sternocleidomastoid, splenius capitis, and trapezius muscles), the abdomen region (rectus abdominis, externus abdominis, longissimus, and iliocostalis muscles), and the lower extremity region (rectus femoris, tibialis anterior, bicep femoris, and medial gastrocnemius muscles). Surface EMG sensors and reflective markers were attached to the volunteer’s skin with stickers, and these stickers’ affixing force was not significantly affected to measure muscle activities^14–17,36^. The EMG signal data were analyzed using EMGworks software (ver. 4.0; Delsys, Inc.). The EMG signal data were sequentially processed by a fourth band-pass filter (10–1000 Hz), rectified using a root-mean-square technique, and normalized based on the measured maximum voluntary contraction (MVC) by each muscle.26 Therefore, all measured muscle activity is presented as %MVC.

### AEB and PSS operations

**Figure 8:**
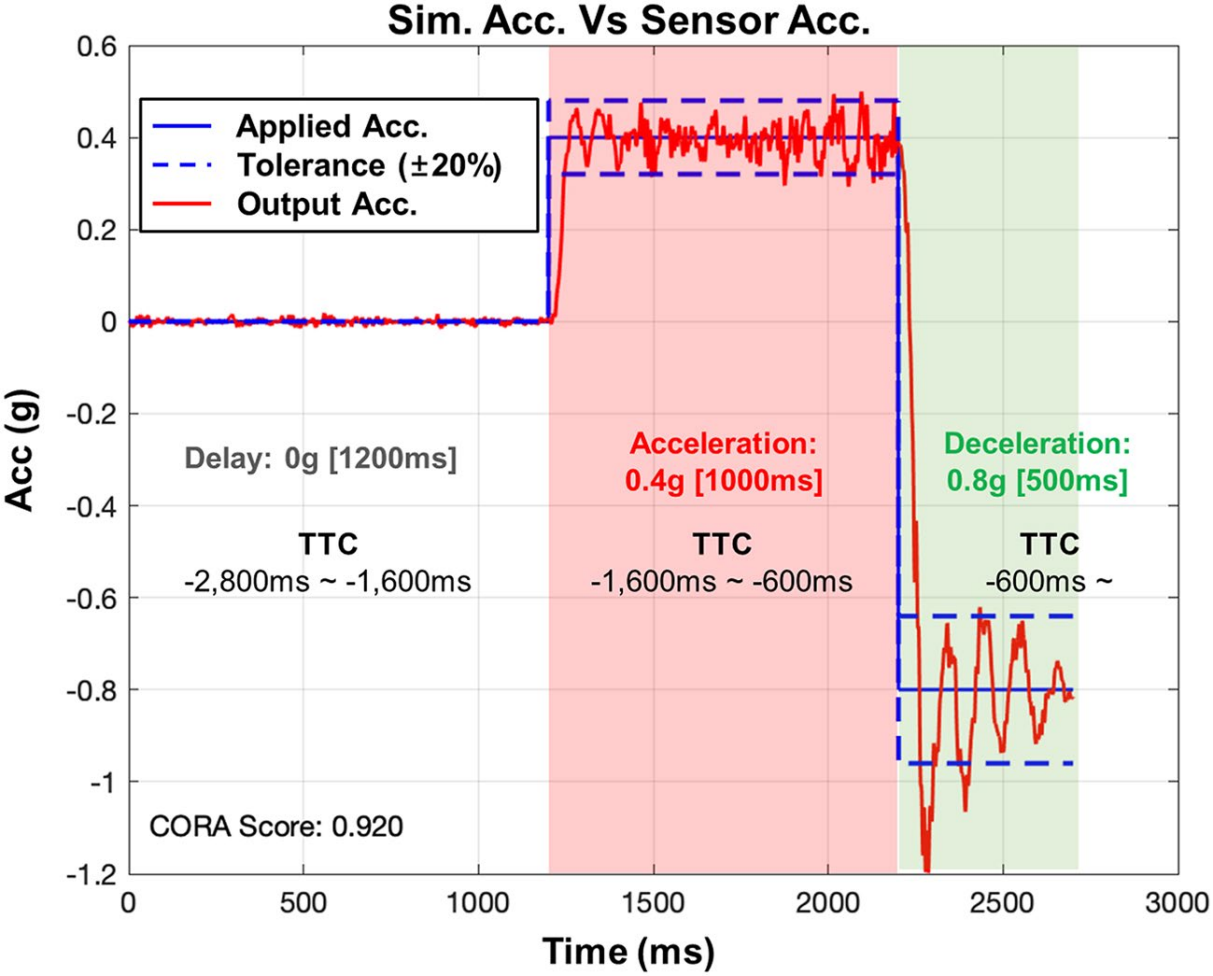
Characteristics of acceleration simulated for AEB operation based on the TTC algorithm. The acceleration simulated for AEB operation was validated by comparison with acceleration measured on the test buck.

A two-stage braking maneuver of a typical vehicle kinematic algorithm was presumed to reproduce AEB operating conditions. According to general vehicle kinematics, at time-to-collision (TTC) 1.6 s, maximal deceleration of 0.4 g and other evasive actions (braking or steering) remain possible^28^. At TTC 0.6 s, with persistent braking from TTC 1.6 s, the collision is considered to be unavoidable and maximal deceleration is increased to 0.8 g^27^. The AEB in this study was designed with a step-function waveform exhibiting acceleration to 0.4 g for 1000 ms and deceleration to 0.8 g for 500 ms (Figure 8). The reliability of simulated input acceleration for reproducing AEB was validated by the correlation and analysis score, in comparison with results obtained using an accelerometer (model 4000A & 4001A accelerometer; TE Connectivity, Schaffhausen, Switzerland) attached to the test buck. The correlation and analysis scale is an objective rating method developed by Gehre et al.^37^, where a minimum score of 0.7 is the criterion for reliability. The correlation and analysis scores of all resultant output acceleration, compared with simulated input, exceeded 0.85. Therefore, the AEB test platform used in the present study was reliable and suitable for reproducing real-world conditions.

The theoretical target of the PSS is to change the OOSP seat to a safer position with the seat back as upright as possible and seat pan moved to the middle position before TTC 1.6 s. However, the operational specification of the electrical seat control system for the PSS is restricted to < 10°/s in the seat recliner and 60 mm/s in the seat pan slider because of the maximum allowed voltage capacity that can be provided to the seat from the battery. Considering these operational restrictions, we presumed that continuing to operate the PSS after TTC 1.6 s would increase the risk of injury to occupants because of posture changes during AEB deceleration. The PSS operation phase was therefore increased by approximately 1 s via TTC 2.6–1.6 s, which is the operating phase of forward collision warning before braking^27,38^.

### Test procedure

The test procedure matrix is shown in Table 2. The initial OOSP seat back angles that may be associated with risk of injury when occupied were the fully reclined seat angle (27°) and half-reclined seat angle (15°). The position was changed via PSS operation to upright by as much as 10°. There were two seat pan moving modes in the PSS: rearward (beginning at 240 mm) to the mid position (179 mm) and forward (beginning at 120 mm) to the mid position (179 mm). Six test conditions were examined to identify the effectiveness of the PSS for augmentation of occupant safety in OOSP and to identify areas for additional improvements: 1) PSS Off with 15° reclined seat angle (PSS_Off15); 2) PSS Off with 27° reclined seat angle (PSS_Off27); 3) PSS On and the seat pan moved rearward to the mid position with 15° reclined seat angle (PSS_On15R); 4) PSS On and the seat pan moved rearward to the mid position with 27° reclined seat angle (PSS_On27R); 5) PSS On and the seat pan moved forward to the mid position with 15° reclined seat angle (PSS_On15F); and 6) PSS On and the seat pan moved forward to the mid position with 27° reclined seat angle (PSS_On27F). Here, five volunteers were randomly selected and assigned to each condition. To obtain the mean measurements and filter out the outliers, tests for each seat configuration were conducted three times for each volunteer. Volunteers were allowed a 30-min break between repeated tests to minimize the effects of muscle fatigue. To simulate feasible conditions when a vehicle is being driven, each volunteer maintained the specified seated posture before the test, with as little tension as possible^32^. To check the relaxed muscle state before the test, sufficient time was provided for the volunteer to adapt to the specified posture; EMG signals were confirmed with monitoring to ensure that they remained stable in the relaxed state.

**Table 2:** PSS test matrix.

Test condition		Seat Pan movement	Seatback angle	Trial counts (volunteers × repeat)	Muscle condition
PSS OFF	PSS-Off15	Rear	15°	15 (5 × 3)	Relaxed
	PSS-Off27		27°	15 (5 × 3)	
PSS ON (Rear to Mid)	PSS-On15R	Rear → Mid	15° → 5°	30 (10 × 3)	
	PSS-On27R		27° → 17°	30 (10 × 3)	
PSS ON (Fore to Mid)	PSS-On15F	Fore → Mid	15° → 5°	15 (5 × 3)	
	PSS-On27F		27° → 17°	15 (5 × 3)	

### Statistical analysis

We applied one-way analysis of variance followed by Bonferroni’s post hoc test to analyze differences in the kinematic characteristics (e.g., forward linear excursion, excursion traces, and rotational angle) among all test conditions. In all analyses, *P* < 0.05 was considered to indicate statistical significance. Adjusted 95% confidence intervals were estimated for all parameters.

### Ethics declarations

All volunteers provided informed consent and the test process was approved by Sejong University Bioethics Committee, Institutional Review Board (IRB number: SJU-2018-001). Volunteers have performed tests in accordance with relevant named guidelines and regulations, and informed consent was obtained from them.

## Data Availability

All datasets generated during the present study are available from the corresponding author.
